# *KRAS* Mutation Variants and Co-occurring PI3K Pathway Alterations Impact Survival for Patients with Pancreatic Ductal Adenocarcinomas

**DOI:** 10.1093/oncolo/oyac179

**Published:** 2022-09-17

**Authors:** Adam C Diehl, Lindsay M Hannan, David B Zhen, Andrew L Coveler, Gentry King, Stacey A Cohen, William P Harris, Veena Shankaran, Kit M Wong, Steven Green, Natasha Ng, Venu G Pillarisetty, Jonathan G Sham, James O Park, Deepti Reddi, Eric Q Konnick, Colin C Pritchard, Kelsey Baker, Mary Redman, E Gabriela Chiorean

**Affiliations:** Division of Medical Oncology, Department of Medicine, University of Washington, Seattle, WA, USA; Fred Hutchinson Cancer Center, Seattle, WA, USA; Division of Medical Oncology, Department of Medicine, University of Washington, Seattle, WA, USA; Fred Hutchinson Cancer Center, Seattle, WA, USA; Division of Medical Oncology, Department of Medicine, University of Washington, Seattle, WA, USA; Fred Hutchinson Cancer Center, Seattle, WA, USA; Division of Medical Oncology, Department of Medicine, University of Washington, Seattle, WA, USA; Fred Hutchinson Cancer Center, Seattle, WA, USA; Division of Medical Oncology, Department of Medicine, University of Washington, Seattle, WA, USA; Fred Hutchinson Cancer Center, Seattle, WA, USA; Division of Medical Oncology, Department of Medicine, University of Washington, Seattle, WA, USA; Fred Hutchinson Cancer Center, Seattle, WA, USA; Division of Medical Oncology, Department of Medicine, University of Washington, Seattle, WA, USA; Fred Hutchinson Cancer Center, Seattle, WA, USA; Division of Medical Oncology, Department of Medicine, University of Washington, Seattle, WA, USA; Fred Hutchinson Cancer Center, Seattle, WA, USA; Division of Medical Oncology, Department of Medicine, University of Washington, Seattle, WA, USA; Fred Hutchinson Cancer Center, Seattle, WA, USA; Fred Hutchinson Cancer Center, Seattle, WA, USA; Fred Hutchinson Cancer Center, Seattle, WA, USA; Department of Surgery, University of Washington, Seattle, WA, USA; Department of Surgery, University of Washington, Seattle, WA, USA; Department of Surgery, University of Washington, Seattle, WA, USA; Department of Laboratory Medicine and Pathology, University of Washington, Seattle, WA, USA; Department of Laboratory Medicine and Pathology, University of Washington, Seattle, WA, USA; Department of Laboratory Medicine and Pathology, University of Washington, Seattle, WA, USA; Brotman Baty Institute for Precision Medicine, Seattle, WA, USA; Fred Hutchinson Cancer Center, Seattle, WA, USA; Fred Hutchinson Cancer Center, Seattle, WA, USA; Division of Medical Oncology, Department of Medicine, University of Washington, Seattle, WA, USA; Fred Hutchinson Cancer Center, Seattle, WA, USA

**Keywords:** KRAS, PI3K, pancreatic cancer

## Abstract

**Background:**

*KRAS* variant alleles may have differential biological properties which impact prognosis and therapeutic options in pancreatic ductal adenocarcinomas (PDA).

**Materials and Methods:**

We retrospectively identified patients with advanced PDA who received first-line therapy and underwent blood and/or tumor genomic sequencing at the University of Washington between 2013 and 2020. We examined the incidence of *KRAS* mutation variants with and without co-occurring *PI3K* or other genomic alterations and evaluated the association of these mutations with clinicopathological characteristics and survival using a Cox proportional hazards model.

**Results:**

One hundred twenty-six patients had genomic sequencing data; *KRAS* mutations were identified in 111 PDA and included the following variants: G12D (43)/G12V (35)/G12R (23)/other (10). PI3K pathway mutations (26% vs. 8%) and homologous recombination DNA repair (HRR) defects (35% vs. 12.5%) were more common among KRAS G12R vs. non-G12R mutated cancers. Patients with KRAS G12R vs. non-G12R cancers had significantly longer overall survival (OS) (HR 0.55) and progression-free survival (PFS) (HR 0.58), adjusted for HRR pathway co-mutations among other covariates. Within the KRAS G12R group, co-occurring PI3K pathway mutations were associated with numerically shorter OS (HR 1.58), while no effect was observed on PFS.

**Conclusions:**

Patients with PDA harboring KRAS G12R vs. non-G12R mutations have longer survival, but this advantage was offset by co-occurring PI3K alterations. The *KRAS/PI3K* genomic profile could inform therapeutic vulnerabilities in patients with PDA.

## Teaser

This retrospective analysis of patients with metastatic and locally advanced pancreatic ductal adenocarcinoma characterizes prognostic or predictive genomic markers for this patient population, with a focus on KRAS mutational variants and their interaction with concurrent molecular alterations.

**Implications for Practice**

In this study, the authors examined the incidence of *KRAS* and co-occurring genetic mutations and assessed their impact on survival outcomes for patients with advanced pancreatic cancer. *KRAS* mutation variants have distinct biological interactions with downstream pathways. Preclinical data suggest that unlike *KRAS G12D* and *G12V*, the *G12R* variant cannot interact with a key protein, PI3K p110α (PIK3CA) and cannot activate its growth-promoting pathway. The data demonstrate biologically and potentially therapeutically relevant differences among cancers with coexisting *KRAS G12R* and *PI3K* pathway alterations and support further investigation of a rational combination of targeted therapies aimed at cancers with specific *KRAS* variants.

## Introduction

Pancreatic cancer is one of the most aggressive malignancies, with more than 60 000 new cases and, despite several multiagent chemotherapies in use since 2011, 47 000 deaths in 2021.^1^ Exome sequencing studies demonstrated alterations in several prevalent core signaling pathways, among which KRAS is almost ubiquitous, affecting more than 90% of pancreatic cancers.^2,3^ Emerging evidence in the last decade suggests that not all *KRAS* mutation variants are equally pathogenic even when they affect the same codon.^4^ Most *KRAS* mutations in pancreatic cancers occur in exon 2 at codon G12, with a lower incidence at codons G13 and Q61. Within codon G12, mutational substitutions occur more frequently at G12D (41-44%), followed by G12V (29-34%), G12R (16-20%), and G12C (1-3%).^5^

Data on the prognostic impact of various *KRAS* variants are conflicting, with some suggesting that the G12R^6-8^ or Q61^9^ variants may be associated with better survival compared to G12D variants, and others showing no effect or an inverse relationship.^10-14^ The molecular underpinnings of these differences are being elucidated, and the disparate ability of *KRAS* variants to activate the PI3K pathway appears to play a central role.^15,16^

KRAS has not been targetable to date, with the exception of the very rare KRAS G12C variant.^17^ Similarly, the downstream effectors PI3K/AKT/mTOR and RAF/MEK/ERK have not been successfully targeted in unselected patients.^18,19^ Despite preclinical evidence suggesting antitumor efficacy from MEK inhibitors in KRAS G12R mutated cancers due to the potential lack of activation of the downstream PI3K targets,^16^ single agent selumetinib showed no benefit among patients with KRAS G12R mutated pancreatic cancers.^20^ A better understanding of the molecular drivers and the consequent dysregulated signaling pathways in pancreatic cancer is critical to the development of targeted therapies and identifying patient subsets most likely to benefit.

We conducted a retrospective analysis of all patients with metastatic and locally advanced pancreatic ductal adenocarcinoma (PDA) who received at least first-line systemic therapy and underwent next-generation sequencing from tumor or blood samples at any point during their follow-up. Our objective was to characterize additional prognostic or predictive genomic markers for this patient population, with a focus on *KRAS* mutational variants and their interaction with concurrent molecular alterations.

## Materials and Methods

### Study Population

All patients with locally advanced or metastatic PDA who were seen at the University of Washington/Fred Hutchinson Cancer Center (UW/FHCC, Seattle, WA) between August 2013 and July 2020, received at least first-line therapy for advanced disease, had somatic genomic sequencing information available, and had any follow up information were included in this retrospective study. Clinical and pathologic information was abstracted from a chart review from the UW/FHCC electronic medical record. The study was approved by the UW Institutional Review Board and conformed to the Declaration of Helsinki.

Data were collected on patient demographics, Eastern Cooperative Oncology Group (ECOG) performance status (PS) at the time of initiating first-line systemic therapy for the advanced disease, genomic sequencing results, treatment history (including prior surgery, systemic therapy for localized or advanced disease, radiation therapy), date of disease progression, and date of death or last follow-up.

### Genomic Sequencing

Genomic sequencing results included only genetic alterations that were adjudicated to be pathogenic by the reporting sequencing platform. No variants of unknown significance were included. The next-generation sequencing (NGS) platforms included FoundationOne (Foundation Medicine, Cambridge, MA) (80.1%), UW-OncoPlex^TM^ (UW, Seattle, WA) (11.1%), Tempus xT/xF (Tempus, Chicago, IL) (3.2%), Guardant 360 (Guardant Health, Redwood City, CA) (2.4%), CellNetix (CellNetix, Tukwila, WA) (0.8%), and Biotheranostics (Biotheranostics, San Diego, CA) (0.8%).

### Statistical Analysis

Overall survival (OS) was defined as the duration from the start of first-line systemic therapy for advanced disease until the date of death from any cause. OS for patients last known to be alive was censored at the date of the last follow-up. Progression-free survival (PFS) was measured from the start of first-line systemic therapy for an advanced disease until the date of progression on first-line systemic therapy or death, whichever came first. PFS for patients last known to be alive and progression-free was censored at the date of the last follow-up.

The associations between binary categorical variables and mutation status were evaluated using the two-tailed Fisher’s exact test. The associations between non-binary categorical variables and mutation status were evaluated using the likelihood-ratio chi-squared test. Two-sided *t*-tests were used to test for associations between continuous variables and mutation status. The distribution of time-to-event outcomes (PFS and OS) was estimated using the method of Kaplan–Meier. Comparisons of distributions were done using a log-rank test. For multivariable modeling, potential confounders, to be included as variables in the model, were identified primarily based on whether they were statistically significant or near significantly (*P* < .15) associated with the variable of interest (KRAS G12R or PI3K pathway mutation status) in bivariate analysis and were thought to be clinically relevant to the outcome being modeled (survival/response). If two variables showed significant collinearity, only one was used in the model. For example, given the co-occurrence of *PI3K* and *SMAD4* mutations within the KRAS G12R mutated group, *SMAD4* mutations were not considered as a separate covariate in the multivariable analysis model. For multivariable models of survival data, hazard ratios were calculated using the Cox proportional hazards model with *P*-values based on the likelihood ratio test. An *a priori* value of *P* < .05 was considered statistically significant. All statistical analyses were performed using JMP software (version 15; SAS institute, Cary, NC).

Variables included in the multivariable model of OS and PFS for the whole cohort included PI3K pathway mutation status (the variable of interest), sex, stage at diagnosis, *ERBB2* amplification status, *BRCA1/BRCA2/PALB2*, KRAS G12R, and *TP53* mutation status. Variables included in the multivariable model of OS and PFS for the KRAS-mutated cohort included KRAS G12R status (variable of interest), stage and ECOG performance status at the start of first-line systemic therapy for advanced disease, type of first-line chemotherapy, *ERBB2* amplification status, homologous recombination DNA repair (HRR), and PI3K pathway mutation status. Variables included in the multivariable model of OS and PFS for the KRAS G12R mutated cohort included PI3K pathway mutation status (the variable of interest), stage at diagnosis and the receipt of both FOLFIRINOX and Gemcitabine/*nab*-Paclitaxel within the first two lines of therapy. Variables included in the multivariable model of OS and PFS for the non-G12R KRAS mutated cohort included the PI3K pathway (the variable of interest) and *BRCA1/BRCA2/PALB2* mutation status.

## Results

We identified 126 patients who met the selection criteria. As of the data cut-off on July 1, 2020, 96 patients died, 25 patients were alive, and 5 patients were censored at the last follow-up. Among patients alive at the last follow-up, the median follow-up duration was 8.1 months (range 2.4-42.2). Next-generation sequencing was performed on tumor tissue for 121 patients (96%) and on circulating tumor deoxyribonucleic acid (ctDNA) for 5 patients (4%).

### Patient Characteristics

Demographic and clinicopathologic characteristics of the patient population for the KRAS wild-type, G12R and non-G12R mutated subgroups are shown in Table 1. For the entire cohort, the median age was 64 (range 25-82), most had metastatic disease (*n* = 109, 86.5%), and received first-line treatment with FOLFIRINOX (51.6%) or with gemcitabine/*nab*-paclitaxel (31.7%). Demographic and clinicopathologic characteristics for patients with cancers with PI3K pathway mutations vs. wildtype in the entire cohort and in the KRAS G12R and non-G12R mutated subgroups are shown in Supplementary Tables S1, S2, and S3.

**Table 1.** Patient and tumor characteristics

	*KRAS* wild type (*n* = 15)	*KRAS* G12R mutation (*n* = 23)	Non-G12R *KRAS* mutations (*n* = 88)^a^	*P*-value^b^
Age, median (range)	60 (32-77)	63 (52-73)	64.5 (25-82)	.52
Sex				.48
Male	8 (47.7%)	9 (39.1%)	43 (48.9%)	
Female	7 (53.3%)	14 (60.9%)	45 (51.1%)	
Race				.83
White	13 (86.7%)	19 (82.6%)	72 (81.8%)	
Black	1 (6.7%)	1 (4.4%)	2 (2.3%)	
Asian	0 (0%)	3 (13.0%)	14 (15.9%)	
Native American	1 (6.7%)	0 (0%)	0 (0%)	
Grade				.46
1 (Well differentiated)	1 (6.7%)	2 (8.7%)	2 (2.3%)	
2 (Moderately differentiated)	4 (26.7%)	8 (34.8%)	31 (35.2%)	
3 (Poorly differentiated)	2 (13.3%)	5 (21.7%)	19 (21.6%)	
No grade assigned	8 (53.3%)	8 (34.8%)	36 (40.9%)	
Primary site				.78
Head/uncinate	6 (40%)	13 (56.6%)	50 (56.8%)	
Body	5 (33.3%)	6 (26.0%)	18 (20.5%)	
Tail	2 (13.3%)	4 (17.4%)	20 (22.7%)	
Indeterminate	2 (13.3%)	0 (0%)	0 (0%)	
Stage at diagnosis				.99
Resectable	1 (6.7%)	5 (21.7%)	22 (25.0%)	
Borderline resectable	0 (0%)	2 (8.7%)	7 (8.0%)	
Locally advanced	1 (6.7%)	3 (13.0%)	10 (11.4%)	
Metastatic	13 (86.7%)	13 (56.6%)	49 (55.6%)	
Prior resection of primary tumor	1 (6.7%)	8 (34.8%)	28 (31.8%)	.81
Stage at first-line systemic therapy for advanced disease				.095
Locally advanced/unresectable	1 (6.7%)	6 (26.1%)	10 (11.4%)	
Metastatic	14 (93.3%)	17 (73.9%)	78 (88.6%)	
ECOG performance status				.090
0	5 (33.3%)	16 (69.5%)	39 (44.4%)	
1	8 (53.3%)	7 (30.5%)	47 (53.4%)	
2	0	0	1 (1.1%)	
Unknown	2 (13.3%)	0	1 (1.1%)	
First-line chemotherapy				.077
FOLFIRINOX	12 (80%)	14 (60.9%)	39 (44.3%)	
Gemcitabine/*nab*-paclitaxel	2 (13.3%)	4 (17.4%)	34 (38.6%)	
FOLFOX	0	3 (13.0%)	2 (2.3%)	
FOLFIRI	0	0	2 (2.3%)	
Gemcitabine	1 (6.7%)	0	5 (5.7%)	
5FU/Liposomal Irinotecan	0	0	1 (1.1%)	
Other	0	2 (8.7%)	5 (5.7%)	
Second-line chemotherapy				.71
FOLFIRINOX	1 (6.7%)	2 (8.7%)	11 (12.5%)	
Gemcitabine/*nab*-paclitaxel	4 (26.7%)	7 (30.4%)	25 (28.4%)	
FOLFOX	0	0	5 (5.7%)	
FOLFIRI	1 (6.7%)	1 (4.4%)	5 (5.7%)	
Gemcitabine	0	0	1 (1.1%)	
5FU/liposomal irinotecan	1 (6.7%)	0	1 (1.1%)	
Other	1 (6.7%)	4 (17.4%)	11 (12.5%)	
None	7 (46.7%)	9 (39.1%)	29 (33.0%)	
FOLFIRINOX and gemcitabine/*nab*-paclitaxel in first and second-line	4 (26.7%)	7 (30.4%)	30 (34.1%)	.81
Genomic alterations				
* TP53*	7 (46.7%)	18 (78.2%)	64 (72.7%)	.79
* SMAD4*	5 (33.3%)	7 (30.4%)	20 (22.7%)	.43
* CDKN2A*	6 (40%)	8 (34.8%)	34 (38.6%)	.81
* ERBB2*	0	3 (13.0%)	1 (1.1%)	**.028**
* *Any PI3K pathway^c^	2 (13.3%)	6 (26.1%)	7 (8.0%)	**.027**
* PIK3CA*	1 (6.7%)	4 (17.4%)	2 (2.3%)	**.016**
* BRCA1/BRCA2/PALB2*	3 (20%)	5 (21.7%)	5 (5.7%)	**.031**
* *Any HRR^d^	3 (20%)	8 (34.8%)	11 (12.5%)	**.025**

Bolded values indicate significance.

*KRAS* non-G12R mutations included *KRAS* G12D (43), *KRAS* G12V (35), *KRAS* Q61 (6), *KRAS* amplification (6).

*P* value is for the statistical test comparing difference between KRAS G12R mutant variants and *KRAS* non-G12R mutant variants. For all categorical variables, a likelihood-ratio chi-squared test or a two-tailed Fisher’s exact test, as appropriate, was the statistical test used to detect significant differences between groups. For all continuous variables, a *t*-test was the statistical test used to detect significant differences between groups.

PI3K pathway mutated genes include: *PIK3CA, AKT2, RICTOR, PTEN, PIK3C2B, PIK3R1.*

HRR mutated genes include: *BRCA1, BRCA2, PALB2, CHEK2, FANCA, ATM.*

Abbreviations: ECOG, Eastern Cooperative Oncology Group; HRR, homologous recombination DNA damage repair.

### Genomic Alterations

**Figure 1.**
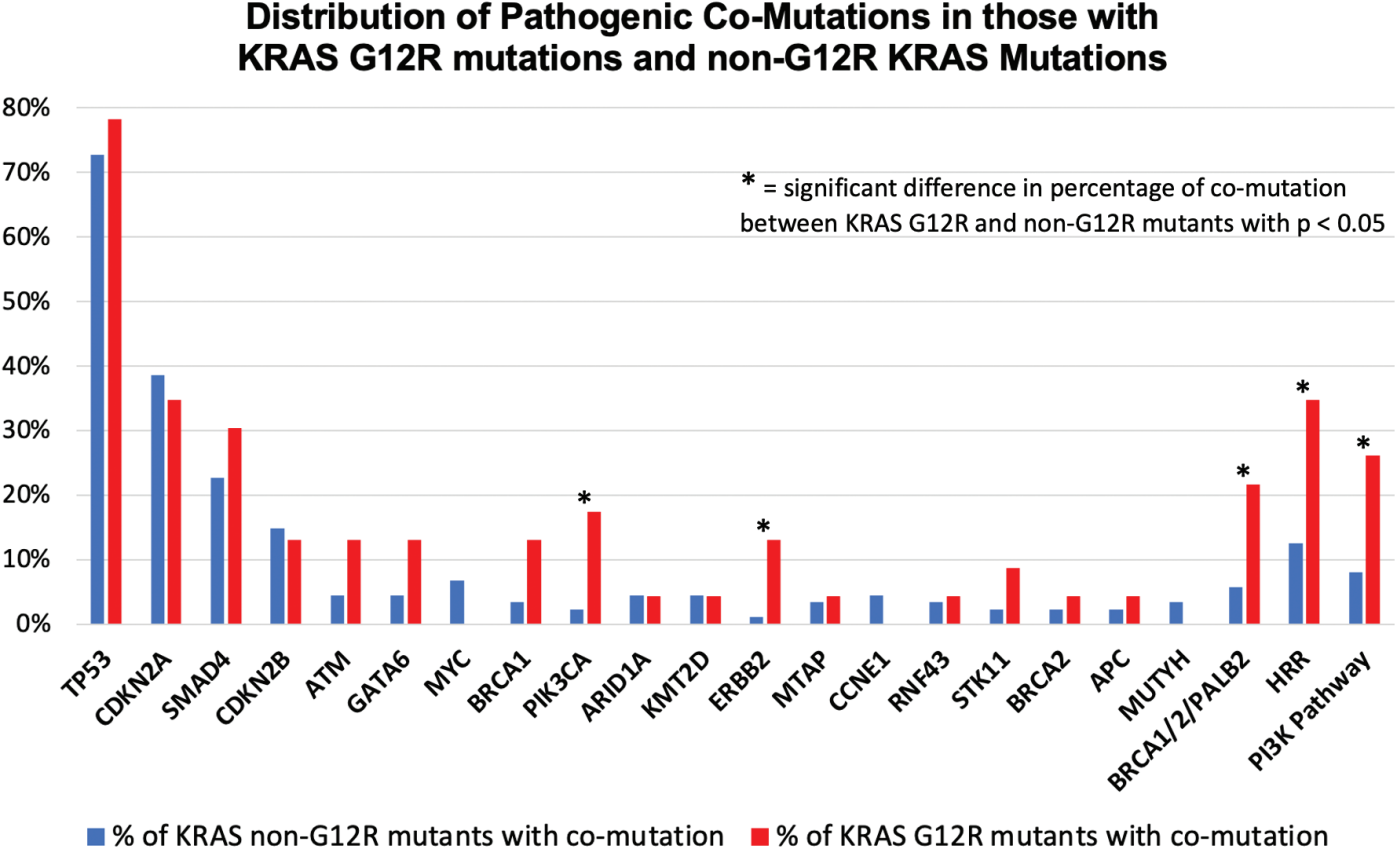
Pathogenic co-mutations in PDA with KRAS G12R and non-G12R mutations. Only the 19 most common co-mutations are shown. Homologous recombination repair (HRR), core HRR (BRCA1, BRCA2, PALB2), and PI3K pathway mutations are shown on the right side.

*KRAS* genetic defects were noted in 111 (88.0%) PDA: *KRAS G12D* (*n* = 43, 38.7% of KRAS mutations), *G12V* (*n* = 35, 31.5%), *G12R* (*n* = 23, 20.7%), *G12C* (*n* = 1, 0.9%), *Q61* (*n* = 6, 5%), and *KRAS* amplification (*n* = 6, 5%). Fifteen (11.9%) cancers harbored 16 PI3K pathway mutations: *PIK3CA* (*n* = 7), *AKT2* and *RICTOR* (*n* = 3 each), *PTEN*, *PIK3C2B*, and *PIK3R1* (*n* = 1 each). Core (*BRCA1/2, PALB2*), and any HRR mutations (*BRCA1/2*, *PALB2*, *CHEK2*, *FANCA*, *ATM*) occurred in 13 (10.3%) and 22 (17.5%) cancers, respectively. The most common genomic alterations in addition to *KRAS* occurred in *TP53* (70.6%), *CDKN2A* (38.1%), and *SMAD4* (25.4%), consistent with data from The Cancer Genome Atlas.^21^Fig. 1 shows the distribution of genomic alterations for KRAS G12R vs. non-G12R mutated cancers. Of note, 15 PDA had KRAS wild-type status. Among these, six cancers had additional genetic alterations: *BRAF* fusions (*n* = 2), and one each with *RET* fusion plus *BRCA2* loss of function mutation, *IDH1* mutation, *RAF1* fusion, and *EML4-NTRK3* fusion.

There were several statistically significant differences in co-occurring mutations between KRAS G12R and non-G12R mutated PDA (Table 1 and Fig. 1). KRAS G12R vs. non-G12R mutated cancers contained significantly more co-mutations in the PI3K pathway (26.1% vs. 8.0%, *P* = .027), and more HRR gene defects (34.8% vs. 12.5%, *P* =.025). KRAS G12R mutated cancers with co-occurring PI3K pathway mutations vs. those without PI3K pathway genetic alterations harbored significantly more *SMAD4* gene defects (83.3% vs. 11.8%, *P* = .0034) (Supplementary Table S2). No significant differences were observed among *KRAS* variants with other genomic alterations, including *TP53*, *CDKN2A*, and *MYC*.

### Correlative Analyses with Clinical and Pathological Characteristics

There were no statistically significant differences in demographic and clinical characteristics between patients with KRAS G12R vs. non-G12R cancers (Table 1, *P* > .05 for all). There were numerically greater proportions of locally advanced vs. metastatic, ECOG PS 0 vs. 1, and first-line treatment with FOLFIRINOX vs. gemcitabine/*nab*-paclitaxel in the KRAS G12R compared to non-G12R mutated groups, but these differences did not reach statistical significance (*P* = .095, .09, and .077, respectively). However, as these associations had *P* < .15, these factors were incorporated into multivariable analyses of survival outcomes.

Within the KRAS G12R mutated group (*n* = 23), patients with cancers harboring PI3K pathway alterations (*n* = 6) vs. none (*n* = 17) were more likely to have metastatic disease at diagnosis (*P* = .098), and to have received both FOLFIRINOX and gemcitabine/*nab*-paclitaxel during their disease course (*P* = .045) (Supplementary Table S2).

### Correlative Analysis with Survival

**Figure 2.**
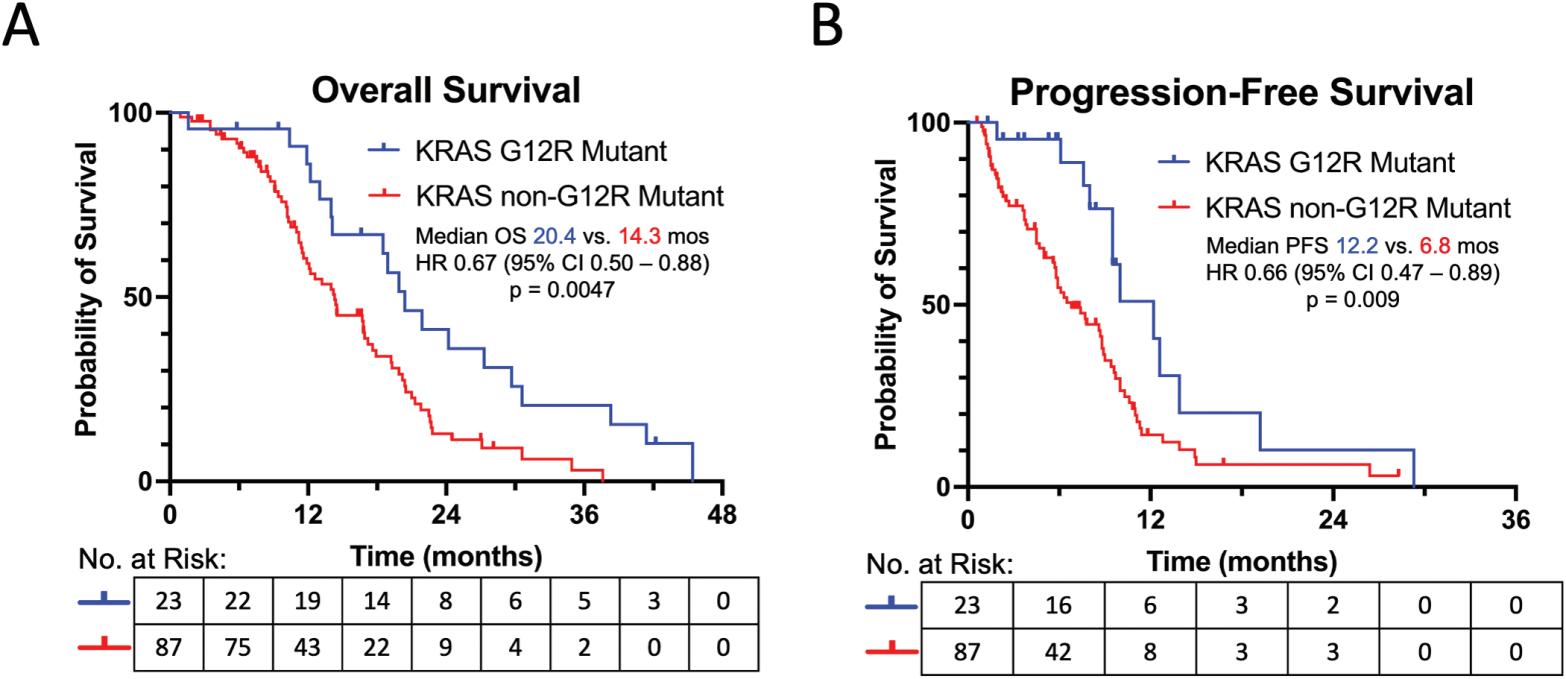
**(A**) Overall survival for KRAS G12R vs. non-G12R mutated PDA. (**B**) Progression-free survival for KRAS G12R vs. non-G12R mutated PDA.

**Figure 3.**
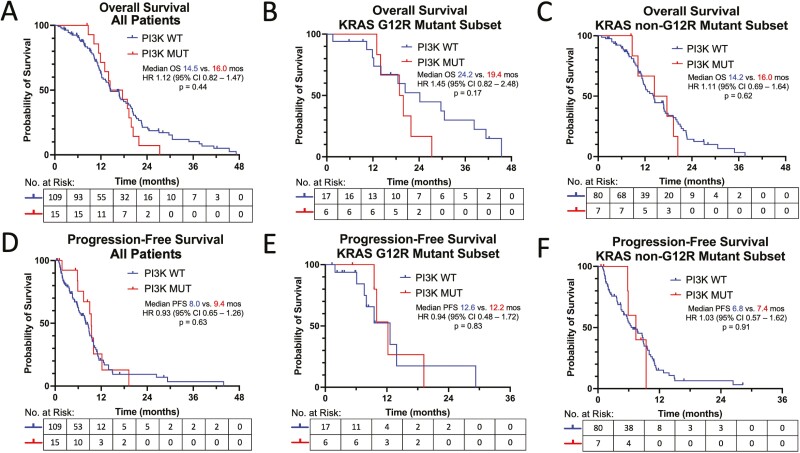
**(A**) Overall survival for PI3K pathway mutated vs. wild type PDA. (**B**) Overall survival for KRAS G12R mutated PDA with vs. without PI3K pathway mutations. (**C**) Overall survival for KRAS non-G12R mutated PDA with vs. without PI3K pathway mutations. (**D**) Progression-free survival for PI3K pathway mutated versus wildtype PDA. (**E**) Progression-free survival for KRAS G12R mutated PDA with vs. without PI3K pathway mutations. (**F**) Progression-free survival for KRAS non-G12R mutated PDA with vs. without PI3K pathway mutations. *P* values based on the log rank test to detect differences in survival.

In the unadjusted analysis, patients with KRAS mutated vs. wild-type PDA had similar survival rates: median OS 14.5 vs. 13.4 months (*P* = .91), and median PFS 8.6 vs. 9.0 months (*P* = .30) with first-line systemic therapies. Patients with KRAS G12R vs. non-G12R mutated cancers had significantly longer median OS (20.4 vs. 14.3 months, *P* = .0047) and PFS (12.2 vs. 6.8 months, *P* = .009) (Fig 2A, 2B). No differences in survival rates were noted among cancers with or without PI3K pathway defects (Fig. 3A, 3D). Within the KRAS G12R mutated group, patients with co-occurring PI3K mutations had numerically shorter OS compared to patients with cancers without PI3K mutations (19.4 vs. 24.2 months, *P* = .17), but had similar PFS (12.2 vs. 12.6 months, *P* = .83) with first-line systemic therapies (Fig. 3B, 3E). Co-occurring PI3K mutations did not influence OS (16.0 vs. 14.2 months, *P* = .62) or PFS (7.4 vs. 6.8 months, *P* = .91) for KRAS non-G12R mutated cancers (Fig 3C, 3F).

No differences in survival were noted among cancers with core or any HRR defects (Supplementary Table S4) compared to HRR-wild type cancers. Within the KRAS G12R mutated subset, survival rates were numerically longer for patients with cancers harboring co-occurring core HRR defects (24.2 vs. 19.9 months, *P* = .60) or any HRR defects (21.9 vs. 19.9 months, *P* = 0.41), although statistical significance was not observed (Supplementary Table S4).

In the adjusted analysis, multivariate Cox proportional hazards models confirmed significantly longer OS (HR = 0.55, *P* = .0008) and PFS with first-line systemic therapies (HR = 0.58, *P* = .0061) in patients with cancers harboring KRAS G12R vs. non-G12R mutations (Table 2). As HRR defects occurred more commonly in KRAS G12R compared to non-G12R mutant cancers, HRR mutational status was included in the multivariable model, but was not significantly associated with OS (*P* = .44) or PFS (*P* = .49). Within the KRAS G12R mutated group, co-occurring PI3K mutations were associated with a trend towards shorter OS (HR = 1.58, *P* = .174).

**Table 2.** Multivariate analysis of survival and response to first-line systemic therapy

Cohort (variables/subgroups)	Patient group, *KRAS*-mutated		All patients		Patient group, *KRAS* G12R-mutated		Patient group, *KRAS* non-G12R-mutated	
	*KRAS* G12R vs. non-G12R variants	*P*-value^c^	PI3K pathway mutated vs. wild-type	*P*-value^c^	PI3K pathway mutated vs. wild-type	*P*-value^c^	PI3K pathway mutated vs. wild-type	*P*-value^c^
OS^a^ Hazard ratio (95% CI)	**0.55** **(0.37-0.79)**	**.0008**	1.03 (0.73-1.45)	.88	**1.58** **(0.81-3.18)**	**.174**	1.05 (0.65-1.56)	.823
PFS^b^ Hazard ratio (95% CI)	**0.58** **(0.38-0.89)**	**.0061**	1.00 (0.66-1.50)	.98	0.78 (0.29-2.13)	.620	1.03 (0.56-1.62)	.922

Bolded values indicate significant or nearly significant P values.

Overall survival (OS) is measured from the start of first-line systemic therapy for advanced disease.

Progression-free survival (PFS) is for first-line systemic therapy for advanced disease.

Likelihood ratio test used to detect significant differences between groups.

## Discussion

Our retrospective analysis explored differences in clinicopathological characteristics and survival in patients with PDA with various oncogenic *KRAS* allele variants with and without co-occurring PI3K pathway and other genomic alterations. Several groups investigated allele-specific signaling in pancreatic cancer cell lines and organoids, and reported that, unlike *KRAS G12D/V* variants, *G12R* has a defective interaction with the key effector PI3K p110α, resulting in decreased PI3K-AKT-mTOR pathway signaling.^16,22^ In addition, KRAS G12R mutated cancers appeared more sensitive to MEK and PI3K inhibitors, preclinically.^16,23^

In our cohort, we found that patients with KRAS G12R mutant PDA had significantly longer survival rates compared to those with non-G12R KRAS mutant tumors, possibly due to deficient PI3K/AKT/mTOR signaling. In addition, likely as an adaptive mechanism to bypass p110α and maintain PI3K signaling output, PI3K pathway mutations were more commonly present in cancers with KRAS G12R mutations compared to other variants. The presence of PI3K pathway mutations did not associate with outcomes in the whole cohort or in patients with non-G12R mutant cancers. However, in the KRAS G12R mutant cohort, concurrent PI3K pathway alterations seemed to offset some of the survival advantages. In addition, in our KRAS G12R mutant cohort, *SMAD4* loss coincided with PI3K pathway mutations. A clear biologic explanation for the collinearity of *SMAD4* loss and PI3K pathway mutations is lacking. Nevertheless, *SMAD4* loss has been linked to non-canonical TGF-β signaling through the MEK/ERK and PI3K/AKT pathways,^24,25^ albeit no association has been described between *SMAD4* loss and OS in advanced stages of pancreatic cancer.^26,27^

Others noted improved prognosis for patients with pancreatic cancer with KRAS G12R mutations vs. alternative variants,^6-8^ but a comprehensive analysis of genomic, transcriptomic, or metabolic dependencies for oncogenic *KRAS* variants and correlations with clinical outcomes and therapeutic targeting has yet to be performed.

KRAS G12R mutated PDA may be differentially targeted with MEK/ERK inhibitors, particularly in the absence of co-occurring PI3K pathway alterations. Preclinically, KRAS G12R mutated cells and organoids have increased sensitivity to MEK/ERK and PI3K inhibitors, with synergistic inhibition from combination MEK/ERK with PI3Kγ or mTOR inhibitors.^16,23,28^ Dual MEK/ERK and PI3K/mTOR pathway blockade may be particularly beneficial given the negative feedback loops activating the parallel pathway when only one is targeted.^29-31^ Biomarker analysis correlating KRAS variants with efficacy from MEK/ERK and/or PI3K/mTOR inhibitors is limited. In a study with the MEK inhibitor cobimetinib plus gemcitabine in 13 patients with pancreatic cancer, all six patients with KRAS G12R mutated cancers achieved disease control, with one partial response and five with stable disease resulting in a median PFS of 6 months. In comparison, those with KRAS G12D/V mutated cancers (*n* = 7) progressed and died within 2 months.^32^ Nevertheless, selumetinib monotherapy had modest activity in refractory KRAS G12R mutated PDA, with median PFS and OS of 3 and 9 months, respectively.^20^ Several larger studies evaluating MEK inhibitors for unselected patients with advanced PDA showed no significant efficacy.^33-36^ Nevertheless, combination strategies with MEK or ERK inhibitors should be studied for pancreatic cancers with KRAS G12R mutations.

Clinical trials with the mTOR inhibitor everolimus alone and in combination with chemotherapy or with molecularly targeted therapies, including EGFR and MEK inhibitors have mostly shown increased toxicity and limited activity.^37-40^PI3K inhibitors were similarly ineffective.^41,42^ There is currently little clinical trial-level biomarker data to determine if there are specific PDA subsets that benefit from PI3K pathway inhibition. Preclinical data suggest that PDA harboring KRAS G12R or PI3K mutations may be more susceptible to PI3K inhibitors.^16,43^ Whether patients with KRAS G12R mutated cancers benefit from PI3K pathway inhibitors is yet unknown.

Dual PI3K/MEK pathway blockade in preclinical models has shown synergism in some studies,^30,44,45^ but clinical trials of unselected patients noted limited efficacy and poor tolerability.^18,46-50^ Whether KRAS G12R mutations with and without co-occurring PI3K mutations could confer specific therapeutic vulnerabilities to combined PI3K/MEK inhibition at lower, more tolerable doses is unknown and deserves further study.

Limitations of our analysis include the retrospective nature of our data collection, the use of a relatively small patient population limited to a single institution, and the heterogeneity of the next-generation sequencing platform used to identify genomic alterations.

## Conclusion

Our analysis of patients with advanced PDA treated with contemporary multiagent chemotherapy supports improved prognosis associated with *KRAS G12R* variant alleles. However, we report a greater incidence of co-occurring PI3K pathway alterations within this subgroup, which appears to offset some of their survival advantages. These exploratory results need to be further validated and support the development of clinical trials in pancreatic cancer to uniquely target *KRAS* mutation variants within context-specific signaling networks.

## Supplementary Material

oyac179_suppl_Supplementary_Table_S1

oyac179_suppl_Supplementary_Table_S2

oyac179_suppl_Supplementary_Table_S3

oyac179_suppl_Supplementary_Table_S4

## Data Availability

The data underlying this article will be shared on reasonable request to the corresponding author.
