# Supplementary material for: KRAS Mutation Variants and Co-occurring PI3K Pathway Alterations Impact Survival for Patients with Pancreatic Ductal Adenocarcinomas
Source: Oncologist. 2022 Sep 17;27(12):1025–33. doi: 10.1093/oncolo/oyac179 (PMC10249424; doi:10.1093/oncolo/oyac179)
Supplement: oyac179_suppl_Supplementary_Table_S1 [file oyac179_suppl_supplementary_table_s1.docx]

| **Table S1: Patient and Tumor Characteristics for PDA with and without PI3K Pathway Mutations** | | | |
| --- | --- | --- | --- |
|  | **PI3K Pathway Mutation (n=15)** | **No PI3K Pathway Mutations (n=111)** | **P-value^a^** |
| **Age, Median (Range)** | 60 (32 - 73) | 64 (25 - 82) | p = 0.24 |
| **Sex** |  |  | **p = 0.028** |
| Male | 3 (20%) | 57 (51.4%) |  |
| Female | 12 (80%) | 54 (48.6%) |  |
| **Race** |  |  | p = 0.12 |
| White | 11 (73.3%) | 93 (83.8%) |  |
| Black | 0 (0%) | 4 (3.6%) |  |
| Asian | 3 (20%) | 14 (12.6%) |  |
| Native American | 1 (6.7%) | 0 (0%) |  |
| **Grade** |  |  | p=0.18 |
| 1 (well differentiated) | 0 (0%) | 5 (4.5%) |  |
| 2 (moderately differentiated) | 3 (20%) | 40 (36.0%) |  |
| 3 (poorly differentiated) | 5 (33.3%) | 21 (18.9%) |  |
| No grade assigned | 7 (46.7%) | 45 (40.5%) |  |
| **Primary Site** |  |  | p = 0.89 |
| Head/Uncinate | 8 (53.3%) | 61 (55.0%) |  |
| Body | 4 (26.7%) | 25 (22.5%) |  |
| Tail | 3 (20%) | 23 (20.7%) |  |
| Indeterminate | 0 (0%) | 2 (1.8%) |  |
| **Stage at Diagnosis** |  |  | **p = 0.026** |
| Resectable | 0 (0%) | 28 (25.2%) |  |
| Borderline Resectable | 2 (13.3%) | 7 (6.3%) |  |
| Locally Advanced | 1 (6.7%) | 13 (11.7%) |  |
| Metastatic | 12 (80%) | 63 (56.8%) |  |
| **Prior Resection of Primary Tumor** | 1 (6.7%) | 36 (32.4%) | p = 0.066 |
| **Stage at First-Line Systemic Therapy**  **for Advanced Disease** |  |  | p = 0.69 |
| Locally Advanced/Unresectable | 1 (6.7%) | 16 (14.4%) |  |
| Metastatic | 14 (93.3%) | 95 (85.6%) |  |
| **ECOG Performance Status** |  |  | p = 0.30 |
| 0 | 10 (66.7%) | 50 (45.0%) |  |
| 1 | 5 (33.3%) | 57 (51.4%) |  |
| 2 | 0 (0%) | 1 (0.9%) |  |
| Unknown | 0 (0%) | 3 (2.7%) |  |
| **First-Line Chemotherapy** |  |  | p = 0.16 |
| FOLFIRINOX | 12 (80%) | 53 (47.7%) |  |
| Gemcitabine/nab-Paclitaxel | 1 (6.7%) | 39 (35.1%) |  |
| FOLFOX | 0 (0%) | 5 (4.5%) |  |
| FOLFIRI | 0 (0%) | 2 (1.8%) |  |
| Gemcitabine | 1 (6.7%) | 5 (4.5%) |  |
| 5FU/Liposomal Irinotecan | 0 (0%) | 1 (0.9%) |  |
| Other | 1 (6.7%) | 6 (5.4%) |  |
| **Second-Line Chemotherapy** |  |  | p = 0.26 |
| FOLFIRINOX | 0 (0%) | 14 (12.6%) |  |
| Gemcitabine/nab-Paclitaxel | 6 (40%) | 30 (27.0%) |  |
| FOLFOX | 1 (6.7%) | 4 (3.6%) |  |
| FOLFIRI | 0 (0%) | 7 (6.3%) |  |
| Gemcitabine | 0 (0%) | 1 (0.9%) |  |
| 5FU/Liposomal Irinotecan | 0 (0%) | 2 (1.8%) |  |
| Other | 3 (20%) | 13 (11.7%) |  |
| None | 5 (33.3%) | 40 (36.0%) |  |
| **Both FOLFIRINOX and Gemcitabine/nab-Paclitaxel in First and Second-Lines** | 6 (40%) | 35 (31.5%) | p = 0.56 |
| **Genomic Alterations** |  |  |  |
| *TP53* | 15 (100%) | 74 (66.7%) | **p = 0.0053** |
| *SMAD4* | 5 (33.3%) | 27 (24.3%) | p = 0.53 |
| *CDKN2A* | 6 (40%) | 42 (37.8%) | p = 1.0 |
| *ERBB2* | 2 (13.3%) | 2 (1.8%) | p = 0.069 |
| *BRCA1/BRCA2/PALB2* | 4 (26.7%) | 9 (8.1%) | **p = 0.049** |
| Any HRR^b^ | 5 (33.3%) | 17 (15.3%) | p = 0.138 |
| *KRAS G12R* | 6 (40%) | 17 (15.3%) | **p = 0.031** |

Abbreviations: PDA (pancreatic ductal adenocarcinoma), HRR (Homologous Recombination DNA Damage Repair)

a) For all categorical variables, a likelihood-ratio chi-squared test or a two-tailed Fisher's exact test, as appropriate, was the statistical test used to detect significant differences between groups. For all continuous variables, a t-test was the statistical test used to detect significant differences between groups.

b) HRR mutated genes include: BRCA1, BRCA2, PALB2, CHEK2, FANCA, ATM.
