# Supplementary material for: KRAS Mutation Variants and Co-occurring PI3K Pathway Alterations Impact Survival for Patients with Pancreatic Ductal Adenocarcinomas
Source: Oncologist. 2022 Sep 17;27(12):1025–33. doi: 10.1093/oncolo/oyac179 (PMC10249424; doi:10.1093/oncolo/oyac179)
Supplement: oyac179_suppl_Supplementary_Table_S2 [file oyac179_suppl_supplementary_table_s2.docx]

| **Table S2: Patient and Tumor Characteristics for KRAS G12R Mutant PDA**  **with and without PI3K Pathway Co-Mutations** | | | |
| --- | --- | --- | --- |
|  | **PI3K Pathway Mutations (n=6)^a^** | **No PI3K Pathway Mutations (n=17)** | **P-value^b^** |
| **Age, Median (Range)** | 63.5 (52 - 73) | 63 (52 - 73) | p = 0.69 |
| **Sex** |  |  | p = 0.34 |
| Male | 1 (16.7%) | 8 (47.1%) |  |
| Female | 5 (83.3%) | 9 (52.9%) |  |
| **Race** |  |  | p = 0.71 |
| White | 5 (83.3%) | 14 (82.3%) |  |
| Black | 0 | 1 (5.9%) |  |
| Asian | 1 (16.7%) | 2 (11.8%) |  |
| Native American | 0 | 0 |  |
| **Grade** |  |  | p = 0.32 |
| 1 (well differentiated) | 0 | 2 (11.8%) |  |
| 2 (moderately differentiated) | 1 (16.7%) | 7 (41.2%) |  |
| 3 (poorly differentiated) | 2 (33.3%) | 3 (17.6%) |  |
| No grade assigned | 3 (50%) | 5 (29.4%) |  |
| **Primary Site** |  |  | p = 0.90 |
| Head/Uncinate | 3 (50%) | 10 (58.8%) |  |
| Body | 2 (33.3%) | 4 (23.5%) |  |
| Tail | 1 (16.7%) | 3 (17.7%) |  |
| Indeterminate | 0 | 0 |  |
| **Stage at Diagnosis** |  |  | p = 0.098 |
| Resectable | 0 | 5 (29.4%) |  |
| Borderline Resectable | 1 (16.7%) | 1 (5.9%) |  |
| Locally Advanced | 0 | 3 (17.7%) |  |
| Metastatic | 5 (83.3%) | 8 (47.0%) |  |
| **Prior Resection of Primary Tumor** | 0 | 8 (47.0%) | p = 0.058 |
| **Stage at First-Line Systemic Therapy**  **for Advanced Disease** |  |  | p = 0.14 |
| Locally Advanced/Unresectable | 0 | 6 (35.3%) |  |
| Metastatic | 6 (100%) | 11 (64.7%) |  |
| **ECOG Performance Status** |  |  | p = 0.62 |
| 0 | 5 (83.3%) | 11 (64.7%) |  |
| 1 | 1 (16.7%) | 6 (35.3%) |  |
| **First-Line Chemotherapy** |  |  | p = 0.30 |
| FOLFIRINOX | 5 (83.3%) | 9 (52.9%) |  |
| Gemcitabine/nab-Paclitaxel | 1 (16.7%) | 3 (17.65%) |  |
| FOLFOX | 0 | 3 (17.65%) |  |
| FOLFIRI | 0 | 0 |  |
| Gemcitabine | 0 | 0 |  |
| 5FU/Liposomal Irinotecan | 0 | 0 |  |
| Other | 0 | 2 (11.8%) |  |
| **Second-Line Chemotherapy** |  |  | p = 0.24 |
| FOLFIRINOX | 0 | 2 (11.8%) |  |
| Gemcitabine/nab-Paclitaxel | 4 (66.6%) | 3 (17.65%) |  |
| FOLFOX | 0 | 0 |  |
| FOLFIRI | 0 | 1 (5.9%) |  |
| Gemcitabine | 0 | 0 |  |
| 5FU/Liposomal Irinotecan | 0 | 0 |  |
| Other | 1 (16.7%) | 3 (17.65%) |  |
| None | 1 (16.7%) | 8 (47.0%) |  |
| **Both FOLFIRINOX and Gemcitabine/nab-Paclitaxel in First and Second-Lines** | 4 (66.7%) | 3 (17.7%) | **p = 0.045** |
| **Genomic Alterations** |  |  |  |
| *TP53* | 6 (100%) | 12 (70.6%) | p = 0.27 |
| *SMAD4* | 5 (83.3%) | 2 (11.8%) | **p = 0.0034** |
| *CDKN2A* | 3 (50%) | 5 (29.4%) | p = 0.62 |
| *ERBB2* | 2 (33.3%) | 1 (5.9%) | P = 0.16 |
| *BRCA1/BRCA2/PALB2* | 2 (33.3%) | 3 (17.7%) | p = 0.58 |
| Any HRR^c^ | 3 (50%) | 5 (29.4%) | p = 0.62 |

Abbreviations: PDA (pancreatic ductal adenocarcinoma), HRR (Homologous Recombination DNA Damage Repair)

a) PI3K pathway mutations were PIK3CA (4), RICTOR (1), PIK32B (1)

b) For all categorical variables, a likelihood-ratio chi-squared test or a two-tailed Fisher's exact test, as appropriate, was the statistical test used to detect significant differences between groups. For all continuous variables, a t-test was the statistical test used to detect significant differences between groups.

c) HRR mutated genes include: *BRCA1, BRCA2, PALB2, CHEK2, FANCA, ATM*.
