# Supplementary material for: KRAS Mutation Variants and Co-occurring PI3K Pathway Alterations Impact Survival for Patients with Pancreatic Ductal Adenocarcinomas
Source: Oncologist. 2022 Sep 17;27(12):1025–33. doi: 10.1093/oncolo/oyac179 (PMC10249424; doi:10.1093/oncolo/oyac179)
Supplement: oyac179_suppl_Supplementary_Table_S3 [file oyac179_suppl_supplementary_table_s3.docx]

| **Table S3: Patient and Tumor Characteristics for non-G12R KRAS Mutant PDA**  **with and without PI3K Pathway Co-mutations** | | | |
| --- | --- | --- | --- |
|  | **PI3K Pathway Mutation (n=7)^a^** | **No PI3K Pathway Mutations (n=81)** | **P-value^b^** |
| **Age, Median (Range)** | 65 (25 - 82) | 62 (50 - 69) | p = 0.66 |
| **Sex** |  |  | p = 0.43 |
| Male | 2 (28.6%) | 41 (50.6%) |  |
| Female | 5 (71.4%) | 40 (49.4%) |  |
| **Race** |  |  | p = 0.59 |
| White | 5 (71.4%) | 67 (82.7%) |  |
| Black | 0 (0%) | 2 (2.5%) |  |
| Asian | 2 (28.6%) | 12 (14.8%) |  |
| Native American | 0 (0%) | 0 (0%) |  |
| **Grade** |  |  | p = 0.75 |
| 1 (well differentiated) | 0 (0%) | 2 (2.5%) |  |
| 2 (moderately differentiated) | 2 (28.6%) | 29 (35.8%) |  |
| 3 (poorly differentiated) | 2 (28.6%) | 17 (21.0%) |  |
| No grade assigned | 3 (42.8%) | 33 (40.7%) |  |
| **Primary Site** |  |  | p = 0.88 |
| Head/Uncinate | 4 (57.1%) | 46 (56.8%) |  |
| Body | 1 (14.3%) | 17 (21.0%) |  |
| Tail | 2 (28.6%) | 18 (22.2%) |  |
| Indeterminate | 0 (0%) | 0 (0%) |  |
| **Stage at Diagnosis** |  |  | p = 0.23 |
| Resectable | 0 (0%) | 22 (27.2%) |  |
| Borderline Resectable | 1 (14.3%) | 6 (7.4%) |  |
| Locally Advanced | 1 (14.3%) | 9 (11.1%) |  |
| Metastatic | 5 (71.4%) | 44 (54.3%) |  |
| **Prior Resection of Primary** | 1 (14.3%) | 27 (33.3%) | p = 0.42 |
| **Stage at First-Line Systemic Therapy**  **for Advanced Disease** |  |  | p = 0.58 |
| Locally Advanced/Unresectable | 1 (14.3%) | 9 (11.1%) |  |
| Metastatic | 6 (85.7%) | 72 (88.9%) |  |
| **ECOG Performance Status** |  |  | p = 0.74 |
| 0 | 4 (57.1%) | 35 (43.2%) |  |
| 1 | 3 (42.9%) | 44 (54.4%) |  |
| 2 | 0 (0%) | 1 (1.2%) |  |
| Not Documented | 0 (0%) | 1 (1.2%) |  |
| **First-Line Chemotherapy** |  |  | p = 0.17 |
| FOLFIRINOX | 5 (71.4%) | 34 (42.0%) |  |
| Gemcitabine/nab-Paclitaxel | 0 (0%) | 34 (42.0%) |  |
| FOLFOX | 0 (0%) | 2 (2.5%) |  |
| FOLFIRI | 0 (0%) | 2 (2.5%) |  |
| Gemcitabine | 1 (14.3%) | 4 (4.9%) |  |
| 5FU/Liposomal Irinotecan | 0 (0%) | 0 (0%) |  |
| Other | 1 (14.3%) | 5 (6.1%) |  |
| **Second-Line Chemotherapy** |  |  | p = 0.73 |
| FOLFIRINOX | 0 (0%) | 11 (13.6%) |  |
| Gemcitabine/nab-Paclitaxel | 1 (14.3%) | 24 (29.6%) |  |
| FOLFOX | 1 (14.3%) | 4 (4.9%) |  |
| FOLFIRI | 0 (0%) | 5 (6.2%) |  |
| Gemcitabine | 0 (0%) | 1 (1.2%) |  |
| 5FU/Liposomal Irinotecan | 0 (0%) | 1 (1.2%) |  |
| Other | 1 (14.3%) | 10 (12.4%) |  |
| None | 4 (57.1%) | 25 (30.9%) |  |
| **FOLFIRINOX and Gemcitabine/nab-Paclitaxel in First and Second-Lines** | 1 (14.3%) | 29 (35.8%) | p = 0.41 |
| **Genomic Alterations** |  |  |  |
| KRAS Variants  *KRAS G12C*  *KRAS G12D*  *KRAS G12V*  *KRAS Q61H, Q61K or Q61L*  *KRAS* Amplification | 0  2 (28.6%)  4 (57.1%)  1 (14.3%)  0 | 1 (1.2%)  41 (50.6%)  31 (38.3%)  5 (6.2%)  6 (7.4%) |  |
| *TP53* | 7 (100%) | 57 (70.4%) | p = 0.18 |
| *SMAD4* | 0 | 20 (24.7%) | p = 0.34 |
| *CDKN2A* | 2 (28.6%) | 32 (39.5%) | p = 0.70 |
| *ERBB2* | 0 | 1 (1.2%) | P = 1.0 |
| *BRCA1/BRCA2/PALB2* | 2 (28.6%) | 3 (3.7%) | **p = 0.049** |
| Any HRR^c^ | 2 (28.6%) | 9 (11.1%) | p = 0.21 |

Abbreviations: PDA (pancreatic ductal adenocarcinoma), HRR (Homologous Recombination DNA Damage Repair)

a) PI3K pathway mutations were AKT2 (3), PIK3CA (2), RICTOR (2), PIK3R1 (1); one patient had both AKT2 and RICTOR mutations

b) For all categorical variables, a likelihood-ratio chi-squared test or a two-tailed Fisher's exact test, as appropriate, was the statistical test used to detect significant differences between groups. For all continuous variables, a t-test was the statistical test used to detect significant differences between groups.

c) HRR mutated genes include: *BRCA1, BRCA2, PALB2, CHEK2, FANCA, ATM*.
