# Supplementary material for: KRAS Mutation Variants and Co-occurring PI3K Pathway Alterations Impact Survival for Patients with Pancreatic Ductal Adenocarcinomas
Source: Oncologist. 2022 Sep 17;27(12):1025–33. doi: 10.1093/oncolo/oyac179 (PMC10249424; doi:10.1093/oncolo/oyac179)
Supplement: oyac179_suppl_Supplementary_Table_S4 [file oyac179_suppl_supplementary_table_s4.docx]

| **Table S4: Univariate Survival Analysis by Core and Any HRR Mutation Status** | | | | | | | | |
| --- | --- | --- | --- | --- | --- | --- | --- | --- |
| Cohort | | | All Patients | | KRAS G12R Mutated Patients | | KRAS non-G12R Mutated Patients | |
|  | | Subgroups | Core^d^  HRR Mutation vs. HRR WT | P-value^e^ | Core^d^  HRR Mutation vs. HRR WT | P-value^e^ | Core^d^  HRR Mutation vs. HRR WT | P-value^e^ |
| Variables | |  |  |  |  |  |  |  |
| OS^a^ | Median (months)  HR^c^  95% CI | | 14.1 vs. 16.7  1.06  0.75 – 1.43 | p = 0.70 | 24.2 vs. 19.9  1.16  0.64 – 1.95 | p = 0.60 | 10.2 vs. 14.5  1.62  0.88 – 2.58 | p = 0.07 |
| PFS^b^ | Median (months)  HR^c^  95% CI | | 10.0 vs. 8.0  0.85  0.57 – 1.19 | p = 0.39 | 13.3 vs. 9.5  0.76  0.38 – 1.44 | p = 0.42 | 7.9 vs. 6.8  1.04  0.51 – 1.71 | p = 0.88 |
|  | Subgroups | | Any  HRR Mutation vs. HRR WT | P-value^e^ | Any  HRR Mutation vs. HRR WT | P-value^e^ | Any  HRR Mutation vs. HRR WT | P-value^e^ |
| Variables |  | |  |  |  |  |  |  |
| OS^a^ | Median (months)  HR^c^  95% CI | | 18.5 vs. 14.5  1.02  0.77 – 1.31 | p = 0.91 | 21.9 vs. 19.9  1.24  0.73 – 2.09 | p = 0.41 | 13.2 vs. 14.4  1.12  0.74 – 1.59 | p = 0.55 |
| PFS^b^ | Median (months)  HR^c^  95% CI | | 10.0 vs. 7.8  0.79  0.57 – 1.06 | p = 0.14 | 12.6 vs. 9.5  0.88  0.46 – 1.64 | p = 0.69 | 9.6 vs. 6.3  0.78  0.48 – 1.13 | p = 0.24 |

Abbreviations: HRR (Homologous Recombination DNA Damage Repair), WT (wild type)

a) Overall survival (OS) is measured from the start of first-line systemic therapy for advanced disease.

b) Progression-Free Survival (PFS) is for first-line systemic therapy for advanced disease.

c) HR and 95% confidence interval derived from univariate Cox proportional hazards model.

d) Core HRR mutations defined as mutations in BRCA1, BRCA2 or PALB2.

e) Log rank test used to detect significant differences between groups.
